# Morphological and phylogenetic analyses of *Weissia* (Pottiaceae) in Türkiye

**DOI:** 10.7717/peerj.20967

**Published:** 2026-03-23

**Authors:** Simge Çizgen Tan, Sezer Okay, Serhat Ursavaş

**Affiliations:** 1 Department of Forest Engineering, Çankırı Karatekin University, Çankırı, Turkey; 2 Vaccine Institute, Hacettepe University, Ankara, Turkey; 3 Department of Forest Engineering, Çankiri Karatekin University, Çankırı, Turkey

**Keywords:** AtpB-rbcL, Bryophytes, Phylogenetics, Plastid markers, PsbA, Rps4, Weissia

## Abstract

The genus *Weissia* is a highly diverse group of moss in the family Pottiaceae. Seven species of *Weissia* were included in this study to provide the first phylogenetic analysis along with a morphological assessment in Türkiye. The taxa studied include *W. longifolia, W. brachycarpa, W. controversa, W. controversa* var*. crispata, W. condensa, W. rutilans,* and* W. wilsonii.* We systematically evaluated morphological traits such as leaf length, seta length, capsule dimensions, and spore characteristics to distinguish closely related taxa. We used three genetic markers: *rps4, psbA*, and *atpB-rbcL* intergenic region to analyze their evolutionary relationship. The results showed that phylogenetic relationships among *Weissia* taxa were marker dependent. *W. brachycarpa* and *W. controversa* var. *crispata* were closely related in the* rps4* tree, but this relationship was not consistent in either *psbA* or *atpB-rbcL* trees. Similarly, *W. condensa* and *W. longifolia* were in different clades in the *psbA* tree, while the former taxon was in its own clade and was somewhat related to *W. brachycarpa* in the *atpB-rbcL* tree. These inconsistencies across markers indicate the need for multi-locus approaches, and future studies using nuclear loci and broader geographic sampling may better resolve cryptic species boundaries in *Weissia*. Our finding of *W. wilsonii* in Bursa northwestern Türkiye a species once thought to be endemic England, supports a broader ecological and geographical range of this species than previously assumed. Together, these findings improve our knowledge of *Weissia* diversity in Türkiye and emphasize the value of integrating morphological and molecular methods in bryophyte systematics and conservation.

## Introduction

Globally, the Pottiaceae family is one of the most diverse in terms of genera and species, accounting for approximately 10% of all moss species (Frey et al., 2006). The genus *Weissia* includes around 90 recognized species worldwide, and 12 of these species (represented by 14 taxa) have been recorded in Türkiye, highlighting the country’s notable contribution to the genus’s diversity. *Weissia* Hedw. subgenus *Astomum* Hampe (Pottiaceae) is known for its short setae, closed capsules, and noticeably different perichaetial leaves, which vary in size, shape, and sometimes direction compared to the regular stem leaves (Callaghan, Bell & Forrest, 2019). *Weissia* exhibits high morphological disparity, especially in the ornamentation of its sporophytes, which is thought to be associated with their survival in different environments such as disturbed soils, temporary habitats, or shady forest floors (Proctor, 2000; Smith, 2004). For instance, species occurring on compacted soils or agricultural fields, such as *W. controversa* and *W. brachycarpa*, develop shorter setae and nearly immersed capsules that reduce desiccation risk, while taxa from shaded or humid habitats, like *W. longifolia*, produce longer setae and more elevated capsules that enhance spore dispersal (Vitt, 1981). These examples illustrate how morphological specialization reflects evolutionary adaptation to thrive in harsh habitats.

Despite its ecological and evolutionary significance, the nomenclature and classification of *Weissia* face challenges due to taxonomic incongruences that rely on either gametophytes or sporophytes features (Stoneburner, 1985). For example, *W. controversa* and *W. brachycarpa* exhibit highly similar gametophytic characters but are distinct in seta length and capsule morphology, leading to inconsistent taxonomic treatments in past studies. In addition, *Weissia* is highly plastic in environments where dehydration, light intensity, and temperature fluctuations frequently occur (Porley, 2013). Such environmental conditions tend to be associated with changes in capsule shape, which challenge the identification of *Weissia* in Turkey (Rose, Kriebel & Sytsma, 2016). For example, in the morphologically similar species *W. brachycarpa, W. controversa*, and *W. longifolia*, perichaetial leaf size and orientation vary considerably across different microhabitats. In *W. controversa*, individuals from compacted or disturbed soils often develop shorter, appressed perichaetial leaves, whereas plants from less disturbed or calcareous substrates may show longer, spreading leaves that overlap with the diagnostic range of *W. brachycarpa*. Comparable variation is also observed in W. longifolia across exposed and shaded sites. Such environmentally driven plasticity blurs morphological boundaries among taxa and has contributed to misidentifications and unstable species delimitation in *Weissia*. While morphological traits have provided important clues for distinguishing *Weissia* taxa, they are often insufficient for resolving closely related or cryptic species. The lack of molecular data of *Weissia* species limits our understanding of the genus’s evolutionary history worldwide (Inoue & Tsubota, 2017), especially in restricted areas like Turkey where the genus is highly diverse and present high plasticity of characters given the different environmental conditions.

Fourteen taxa (12 species) of *Weissia* genus have been identified in Türkiye, including the critically endangered *Weissia wilsonii* D.A. Callaghan, recently added to Türkiye’s bryophyte flora (Ursavaş & Keçeli, 2019). While morphological studies have provided valuable insights into these taxa, comprehensive analyses integrating both morphological and molecular data are missing. This knowledge gap limits our understanding of the phylogenetic relationships and ecological adaptations of *Weissia* species in Türkiye. Furthermore, the potential conservation implications for these species remain underexplored.

Several species within *Weissia*, particularly *W. brachycarpa, W. controversa*, and *W. longifolia*, exhibit overlapping morphological characters that complicate species delimitation. These taxa share similar gametophytic traits, including leaf shape and cell dimensions, while showing environmentally driven variation in perichaetial leaf size and orientation across different microhabitats. For example, *W. controversa* populations from compacted or disturbed soils often develop shorter, appressed perichaetial leaves, whereas individuals from less disturbed substrates may display longer, spreading leaves overlapping with the diagnostic range of *W. brachycarpa*. Comparable plasticity has also been observed in W. longifolia across shaded and exposed sites. Such morphological overlaps and plasticity have historically led to misidentifications and inconsistent taxonomic treatments within the genus (Smith, 2004; Zander, 1993).

This study aims to address unresolved taxonomic challenges in the genus *Weissia* by presenting a comparative assessment of selected Turkish taxa using both morphological and plastid phylogenetic data. Specifically, we aim to (i) quantify morphological variation across seven representative taxa, (ii) evaluate the phylogenetic relationships among these taxa based on three chloroplast markers (*rps4, psbA*, and *atpB-rbcL*), and (iii) test the congruency between morphological differentiation and molecular divergence among closely related taxa.

We hypothesize that morphologically similar taxa (*e.g.*, *W. brachycarpa*, *W. controversa*, and *W. longifolia*) represent distinct evolutionary lineages despite overlapping diagnostic traits, reflecting either recent speciation events or historical misclassification.

By integrating both datasets, this research explicitly addresses the following questions:

1. Do morphological traits reliably distinguish closely related *Weissia* taxa in Türkiye?
2. How consistent are the phylogenetic relationships inferred from different plastid markers?
3. To what extent do molecular results support or challenge current taxonomic-based classification?

The outcomes of this study will refine species boundaries, improve taxonomic resolution, and inform conservation priorities for Turkish bryophyte flora.

## Materials and Methods

### Plant samples

A total of 44 specimens representing seven *Weissia* taxa were analyzed in this study. For each taxon, four to five individuals were selected to ensure morphological variation of natural populations. Specimens of *W. wilsonii, W. condensa,* and *W. rutilans* were collected from Bursa-Karacabey (between Bayramdere and Bogaz villager’s locality: 40°21′45″N, 28°25′07″E) in May 2017 during field surveys by Prof. Dr. Serhat Ursavaş. Similarly, *W. brachycarpa* and *W. controversa* var. *crispata* were collected from İğneada Longoz Forests National Park, Kırklareli (Mert Lake locality: 41°52′04″N, 27°58′41″E) in June 2016. One *W. longifolia* specimen was collected from Alpsarı Pond, Çankırı (40°46′18″N, 33°48′50″E) in April 2015.

In addition to these field-collected samples, we also included herbarium specimens provided by colleagues to increase geographic representation and variability of morphological traits (see Table 1 for detailed voucher information). *W. rutilans* and *W. controversa* samples were kindly supplied by Prof. Dr. Mesut Kırmacı (Aydın Adnan Menderes University), and one *W. longifolia* specimen was provided by Prof. Dr. Nevzat Batan (Karadeniz Technical University). All specimens were preserved as dried herbarium material and are stored in the personal herbarium of Prof. Dr. Serhat Ursavaş in the Faculty of Forestry at Çankırı Karatekin University.

**Table 1:** *Weissia* taxa included in the morphological, taxonomic, and molecular analyses, indicating PCR/sequencing success, marker coverage, and corresponding herbarium voucher numbers.

**Species**	**Morphological analysis**	**Taxonomic key**	**PCR/sequencing**	***rps4***	***psbA***	***atpB-rbcL***	**Voucher no**
*W. longifolia*	✓	✓	✗	✗	✗	✗	ZNG780
*W. brachycarpa*	✓	✓	✓	✓	✓	✓	U2293
*W. controversa*	✓	✓	✗	✗	✗	✗	ZNG2030
*W. controversa* var*. crispata*	✓	✓	✓	✓	✓	✓	U2294
*W. condensa*	✓	✓	✗	✗	✗	✗	U3072
*W. rutilans*	✓	✓	✗	✗	✗	✗	AYDN1552
*W. wilsonii*	✓	✓	✗	✗	✗	✗	U3090

**Notes.**

Voucher No: ZNG Zonguldak Bülent Ecevit University Herbarium AYDN Aydın Adnan Menderes University Herbarium U personal herbarium of Ursavaş PCR polymerase chain reaction

PCR/sequencing success indicates taxa for which at least one plastid marker yielded usable sequence data and was included in phylogenetic analyses.

### Morphological assessment

We measured key morphological traits of seven species of *Weissia* found in Türkiye: *W. longifolia, W. brachycarpa, W. controversa, W. controversa* var*. crispata, W. condensa, W. rutilans,* and *W. wilsonii.* We analyzed plant height (mm), presence of branches, the length and width of the perichaetial leaves (mm), the length of the seta (mm), the length and width of the capsule (mm), the ratio of the capsule’s width to its length, the presence and length of the operculum and peristome (µm), the size and surface texture of the spores (µm), and the dimensions of cells in different parts of the leaves (µm). These traits were selected based on widely used bryological identification keys (Braithwaite, 1887; Dixon & Jameson, 1924; Crundwell & Nyholm, 1972; Smith, 2004; Frey & Stech, 2009). We measured four to eight samples of each species using a light microscope (Olympus CX31; Olympus, Hachioji, Japan) and calculated the average values of each trait. Because we had a small number of samples, we didn’t do formal statistical tests, but the descriptive data help show the differences between the species. Due to limited sample sizes, formal statistical comparisons were not applied; however, the descriptive data support taxonomic delimitation and provide diagnostic characters for distinguishing closely related taxa. Voucher specimens for each species are deposited in different herbaria, including Zonguldak Bülent Ecevit University Herbarium (ZNG), Aydın Adnan Menderes University Herbarium (AYDN), and the personal herbarium of Ursavaş (U).

### Taxonomic key

The identification key was constructed using a dichotomous system, in which pairs of contrasting morphological traits are presented sequentially to guide species identification. This two-part approach, widely applied in bryological taxonomy, allows consistent separation of taxa based on diagnostic characters such as seta length, perichaetial leaf size, capsule depth, and spore ornamentation. Characters were selected according to their interspecific consistency and diagnostic reliability across specimens, supported by comparisons with standard bryophyte floras and identification manuals (*e.g.*, Smith, 2004; Frey & Stech, 2009).

### DNA extraction and sequencing

#### DNA isolation

We extracted total genomic DNA from five specimens of *Weissia*: *W. longifolia, W. brachycarpa, W. controversa* var. *crispata, W. condensa*, and *W. rutilans*; using a modified version of the cetyltrimethylammonium bromide (CTAB) method described by Suzuki et al. (2013). The modifications included extended incubation at 65 °C for 90 min (instead of 60 min) and the addition of 2% polyvinylpyrrolidone (PVP) to the extraction buffer to reduce phenolic compounds that commonly accumulate in bryophyte tissues and interfere with DNA extraction and downstream enzymatic reactions. Using liquid nitrogen in a sterile mortar and pestle, we ground fresh or dried plant material (approximately 20 mg) into a fine powder. The finely ground plant material was transferred into a microcentrifuge tube containing 500 µL of CTAB extraction buffer (2% CTAB, 100 mM Tris–HCl pH 8.0, 20 mM EDTA, 1.4 M NaCl, and 2% β-mercaptoethanol) pre-heated to 65 °C.

We incubated the mixture at 65 °C for 30 min, occasionally gently mixing it to ensure cell lysis. After the incubation period, we added an equal volume of chloroform:isoamyl alcohol (24:1) and centrifuged the mixture at 12,000 g for 10 min. We carefully transferred the aqueous phase to a new tube and precipitated the DNA by adding an equal volume of ice-cold isopropanol. We centrifuged the tube again at 12,000 g for 15 min to pellet the DNA.

The remaining DNA pellet was washed with 70% ethanol, dried in the air, and then mixed again in 50 µL of TE buffer (10 mM Tris–HCl, 1 mM EDTA, pH 8.0). We assessed DNA purity and concentration using a NanoDrop spectrophotometer and agarose gel electrophoresis. Extracted DNA samples were stored at −20 °C until further use.

Total genomic DNA was extracted from herbarium specimens of five *Weissia* taxa (*W. longifolia*, *W. brachycarpa, W. controversa* var. *crispata, W. condensa*, and *W. rutilans*). Although all taxa were subjected to DNA extraction and polymerase chain reaction (PCR) amplification, clear and reproducible bands were obtained only for *W. brachycarpa* and *W. controversa* var. *crispata*. For the remaining taxa, PCR amplification did not yield usable bands, and therefore these species were excluded from subsequent molecular phylogenetic analyses.

#### PCR amplification and sequencing

Three phylogenetic markers, intergenic region of ATP synthase and ribulose-1,5-bisphosphate carboxylase/oxygenase large subunit (*atpB-rbcL*) genes, ribosomal protein S4 (*rps4*) gene and photosystem II protein gene D1 (*psbA)* gene were amplified using PCR. The PCR mixture consisted of 1X PCR buffer, 5 µL MgCl_2_, 1 mM dNTP mix, 1 µL *Taq* DNA polymerase (all from Fermentas), 1 µL of both forward and reverse primers (Table 2), and 2 ng template DNA. The final volume was adjusted to 50 µL with dH_2_O. PCR conditions were as follows (40 cycles): Initial denaturation at 94 °C for 15 min, denaturation at 94 °C for 1 min, annealing at 50 °C for 1 min, extension at 72 °C for 1.5 min and final extension at 72 °C for 10 min (Inoue & Tsubota, 2014; Shaw, Szövényi & Shaw, 2011).

**Table 2:** Primers used in this study for PCR amplifications.

**DNA marker**	**Primer** **name**	Nucleotide sequence ^*^	**Amplicon length (bp)**	**Species sequenced**
*atpB*-*rbcL*	atpB-1	5′-acatckartackggaccaataa-3′	550	*W. longifolia*, *W. brachycarpa*, *W. controversa* var*. crispata*, *W. condensa*
	rbcL-1	5′-aacaccagctttraatccaa-3′	550	*(same as above)*
*rps4*	rps5	5′-atgtcccgttatcgaggacct-3′	600	*W. longifolia*, *W. brachycarpa*, *W. controversa* var*. crispata*
	trnAS	5′-taccgagggttcgaatc-3′	600	*(same as above)*
*psbA*	501F	5′-tttctcagacggtatgcc-3′	1,144	*W. brachycarpa*, *W. controversa* var*. crispata*, *W. condensa*
	trnHR	5′-gaacgacgggaattgaac-3′	1,144	(same as above)

**Notes.**

* K is IUPAC degenerate nucleotide code for G or T.

#### Sequencing

We visualized the amplified PCR products on a 1% agarose gel, stained with ethidium bromide, to confirm successful amplification. We excised and purified bands of the expected size using a QIAquick Gel Extraction Kit (Qiagen, Hilden, Germany) and performed Sanger sequencing using Atlas Biotechnologies Laboratory (Ankara, Türkiye).

The resulting sequences were aligned using ClustalW, and phylogenetic analyses were performed in MEGA X using the Neighbor-Joining method. The sequences generated in this study were deposited in GenBank under the following accession numbers: PQ821690 –PQ821691 for rps4, PQ821692 –PQ821693 for psbA, and PQ821694 –PQ821695 for atpB–rbcL (*W. brachycarpa* and *W. controversa* var. *crispata*). Although PCR amplification was attempted for *W. longifolia*, *W. condensa*, and *W. rutilans*, usable sequence data could not be obtained for these taxa due to poor read quality or failure during sequencing; therefore, they were excluded from the phylogenetic analyses.

### Phylogenetic analysis

Sequence alignment and tree construction: We used ClustalW to align DNA sequences obtained from PCR amplification, making manual adjustments to ensure accuracy. We then used the aligned sequences to construct phylogenetic trees using the Neighbor-Joining (NJ) method, which was implemented in MEGA X software. We calculated genetic distances using the Kimura 2-parameter model and evaluated branch support through 1,000 bootstrap replicates to build the phylogenetic tree.

We used *Scleropodium cespitans* as an outgroup to root the phylogenetic trees, similar to previous studies (Cox et al., 2010; Stech & Frey, 2008).

## Results

### Morphology and ecology

**Figure 1:**
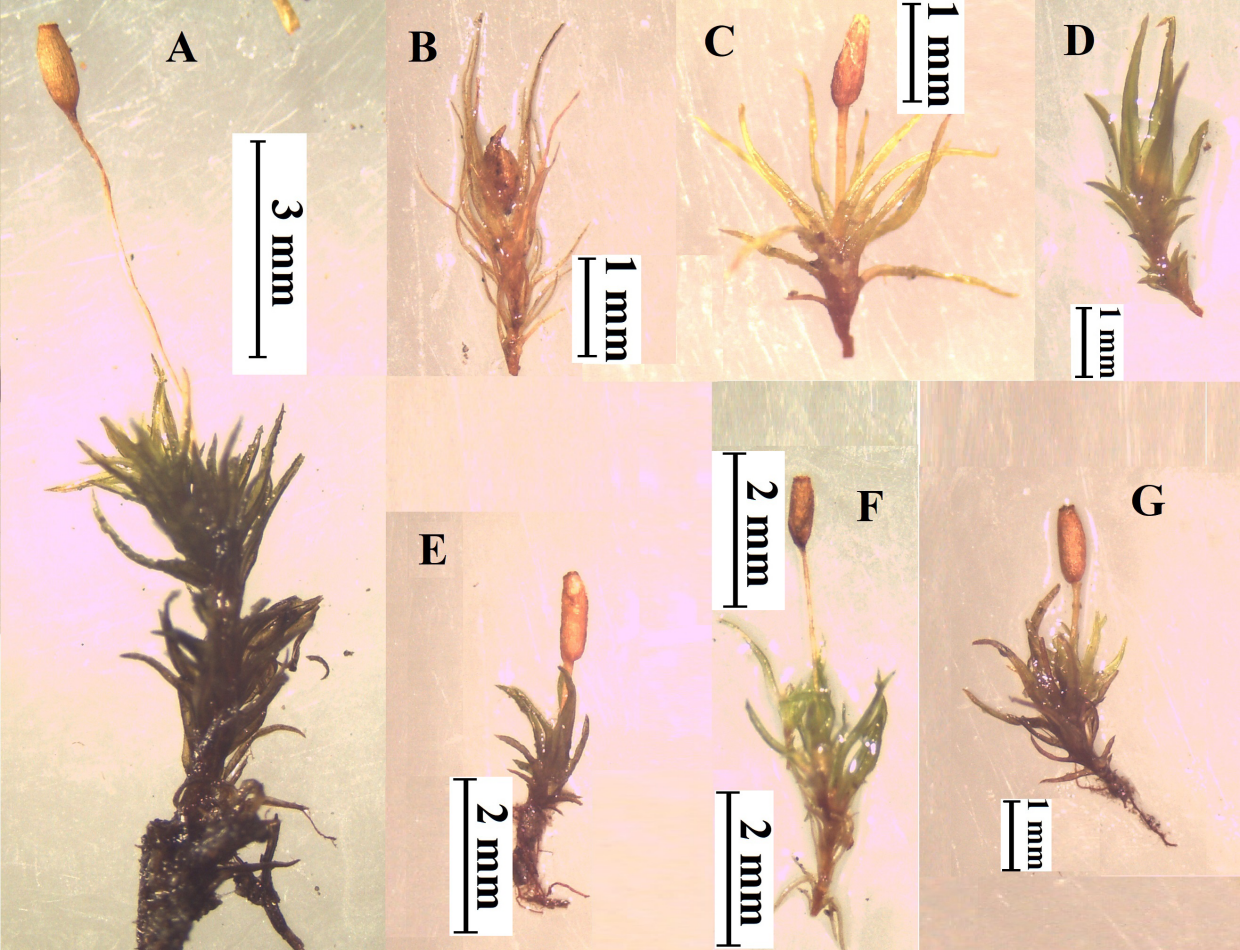
Wet habit of *Weissia* species observed under stereomicroscope ((A) *W. rutilans*, (B) *W. longifolia*, (C) *W. brachycarpa*, (D) *W. wilsonii*, (E) *W. condensa*, (F) *W. controversa*, (G) *W. controversa* var. *crispata*, Photo credit: Serhat URSAVAŞ).

We analyzed the morphological characteristics of seven taxa of *Weissia* identified in Türkiye, including *W. longifolia, W. brachycarpa, W. controversa, W. controversa* var. *crispata, W. condensa, W. rutilans,* and *W. wilsonii* (Fig. 1). We observed consistent morphological differences among species, particularly in leaf length, the shape and size of perichaetial leaves, capsule orientation, and the dimensions of leaf cells (basal, median, and apical). Representative illustrations and microscopic photographs of these diagnostic traits are provided in Fig. 1 to support the observed variation.

For instance, *W. rutilans* exhibited the longest leaves among the studied taxa (mean: 2.5 mm), while *W. wilsonii* displayed the longest leaf basal cells (mean: 85 µm). *W. brachycarpa* and *W. controversa* are frequently confused due to their similar appearance yet can be differentiated by seta length and capsule orientation. For instance, *W. controversa* has longer setae (3–4 mm) and capsule length (1–1.5 mm), whereas *W. brachycarpa* shows shorter setae (1.5–3 mm) and capsule length (1−1.5 mm). Despite the absence of statistical analyses due to the limited sample size, the parameters used in this study serve as useful diagnostic features supporting species delimitation (Table 3).

**Figure 2:**
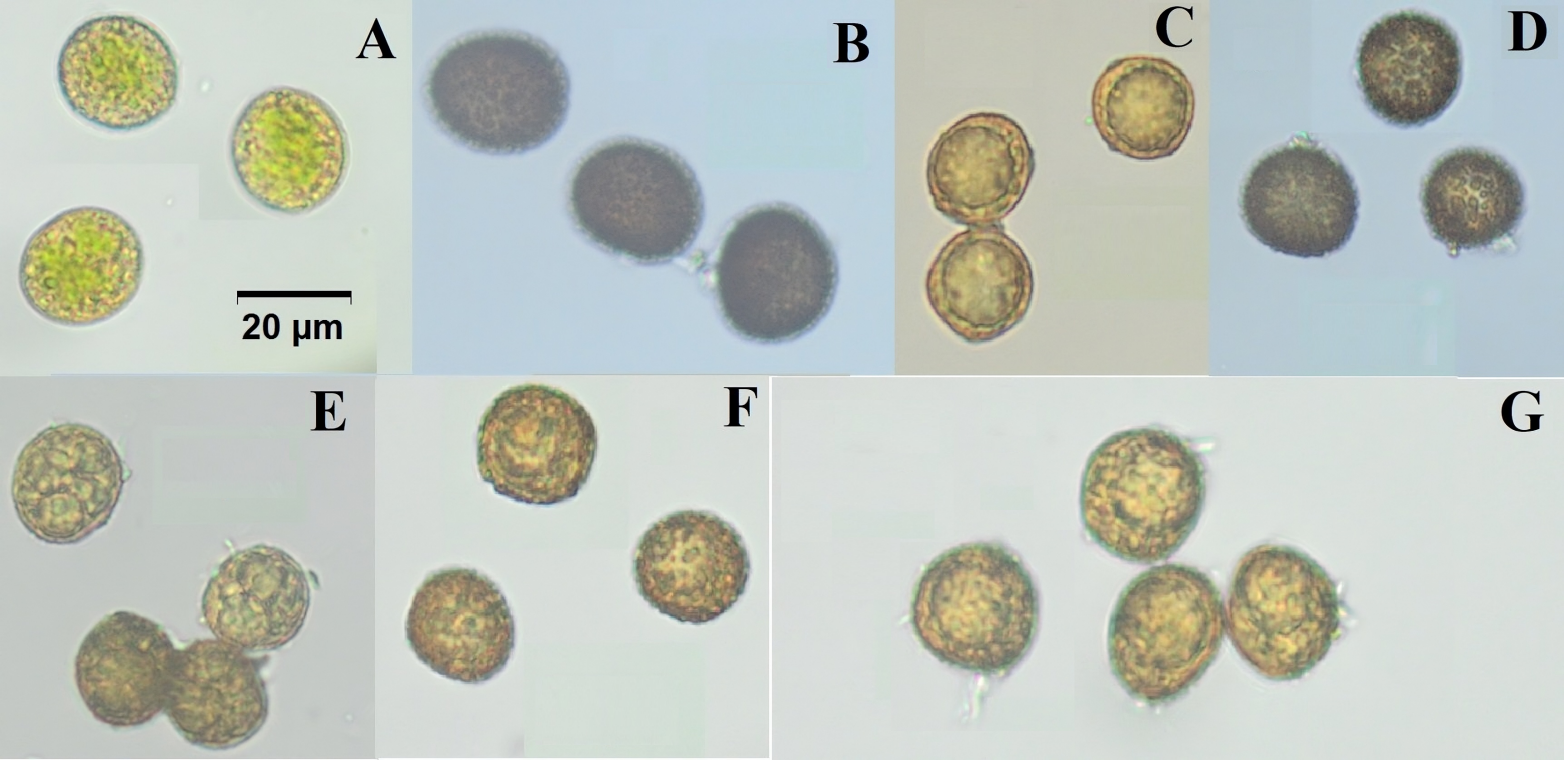
Microscopic images of spores from Weissia species ((A) *W. rutilans*, (B) *W. longifolia*, (C) *W. brachycarpa*, (D) *W. wilsonii*, (E) *W. condensa*, (F) *W. controversa*, (G) *W. controversa* var. crispata). All images are at the same scale; scale bar = 20 µm (shown in panel A). Photo credit: Serhat Ursavaş.

These physical features, particularly spore morphology, while often difficult to observe in nature, are important for accurately identifying species, especially in groups with similar reproductive traits (Fig. 2). A comparative summary of these diagnostic morphological traits is provided in Table 3. In our material, spore ornamentation was useful in distinguishing some taxa, such as *W. rutilans* and *W. wilsonii*, whereas in closely related groups like *W. controversa* and *W. controversa* var. *crispata* it showed little diagnostic value (Can Gözcü, Uyar & Ceter, 2018). *Weissia* species examined in this study exhibit remarkable ecological plasticity, as reflected by their ability to colonize contrasting substrates and microhabitats. For example, *W. longifolia* thrives on exposed rocky slopes with limited moisture, while *W. brachycarpa* occurs on compacted agricultural soils subjected to disturbance. Similarly, *W. controversa* var. *crispata* was found on calcareous cliffs where substrate chemistry influences growth, whereas *W. condensa* occupies shaded forest edges with higher humidity. These observations demonstrate the capacity of *Weissia* taxa to adapt morphologically and physiologically to heterogeneous environmental conditions. These plants help the ecosystem by holding soil together with their thick mats, which prevents erosion and creates small areas that keep moisture and support other small plants or microorganisms.

**Table 3:** Morphological comparison of *Weissia* taxa examined in this study.

**Plant parts**	***Weissia longifolia***	***Weissia brachycarpa***	***Weissia rutilans***	***Weissia condensa***	***Weissia controversa***	***Weissia controversa*** ** var.** ***crispata***	***Weissia wilsonii***
Plant length (mm)	1–5	3–8	**3–15**	3–6	2–8	2–7	3–6
Presence of branching	None	None	None or sometimes branching is present at the tips.	None	Sometimes branching is present at the bottom.	Sometimes branching is present at the bottom.	Sometimes branching is present at the tips.
Leaf length (mm)	1–1.5	1–2	**2–2.5**	1–2	1–1.5	1–2	0.8–2
Perichaetial leaf length (mm)	**2–5**	2–3	2–3	1.5–3	1.5–2	2–3	2–3.5
Ratio of stem leaf length to perichaetial leaf length (mm)	2–5	**0.5–0.7**	1–1.5	1–3	1–2	2–3	1–4
Seta Length (mm)	0.5–1.2	1.5–3	**5–8**	2–4	3–4	1.5–2	0.2–0.8
Capsule Length (mm)	0.5–1	1–1.5	1–1.5	0.7–1	1–1.5	1	**0.5–0.8**
Capsule Width (mm)	0.2–0.5	0.3	0.5	0.4	0.2–0.5	0.3	0.2–0.5
Ratio of urn width to urn length (mm)	1–2.5	**4–5**	2–3	2	**2–7**	3	2–4
Operculum length (mm)	0.2–0.4	0.5	Absent	Absent	0.5	Absent	0.2
Peristome Length (μm)	Absent	Absent	50–70	Absent	**45–100**	Absent	Absent
Spores (μm)	15–25	20–25	16–25	15–25	15–20	16–22	14–20
Spore papillosity	Papillose	Smooth to Slightly papillose	Smooth to Slightly papillose	Smooth to Slightly papillose	Papillose	Smooth	Papillose
Leaf medial cell (μm)	**8–13** **X** **4–8**	6–10 X 4–6	8–12 X 7–10	6–9 X 6–9	5–9 X 5–8	6–10 X 4–6	8 X 5
Leaf basal cell (μm)	20–45 X 7–12	30–40 X 10–18	25–40 X 8–19	18–45 X 7–18	16–45 X 9–14	25–55 X 6–12	**40–85** **X** **10–23**
Leaf apical cell (μm)	**16–28** **X** **4–6**	16–24 X 3–6	14–20 X 5–8	6–12 X 3–5	10–16 X 5–8	11–20 X 5–8	14–19 X 5–8
Leaf base costa thickness (μm)	40–80	36–50	**55–80**	**55–85**	30–50	60–70	40–65

**Notes.**

Bolded values indicate key distinguishing morphological characteristics among the *Weissia* taxa.

*W. wilsonii* was discovered on a shaded, moist, clay-rich bank in Bursa province (northwestern Türkiye), co-occurring with *Tortella tortuosa* and *Fissidens taxifolius*. This record, expanding the species’ known distribution beyond the British Isles, indicates a broader ecological tolerance than previously assumed and suggests overlooked refugial populations in Eurasian Forest ecosystems.

### Phylogenetic analysis

This is the first study to investigate the evolutionary relationship among *Weissia* species in Türkiye using three specific genetic markers: *rps4, psbA*, and the *atpB-rbcL* intergenic region. We analyzed six species and one subspecies: *W. brachycarpa, W. controversa, W. controversa* var. *crispata*, *W. condensa, W. rutilans, W. longifolia*, and *W. wilsonii*.

**Figure 3:**
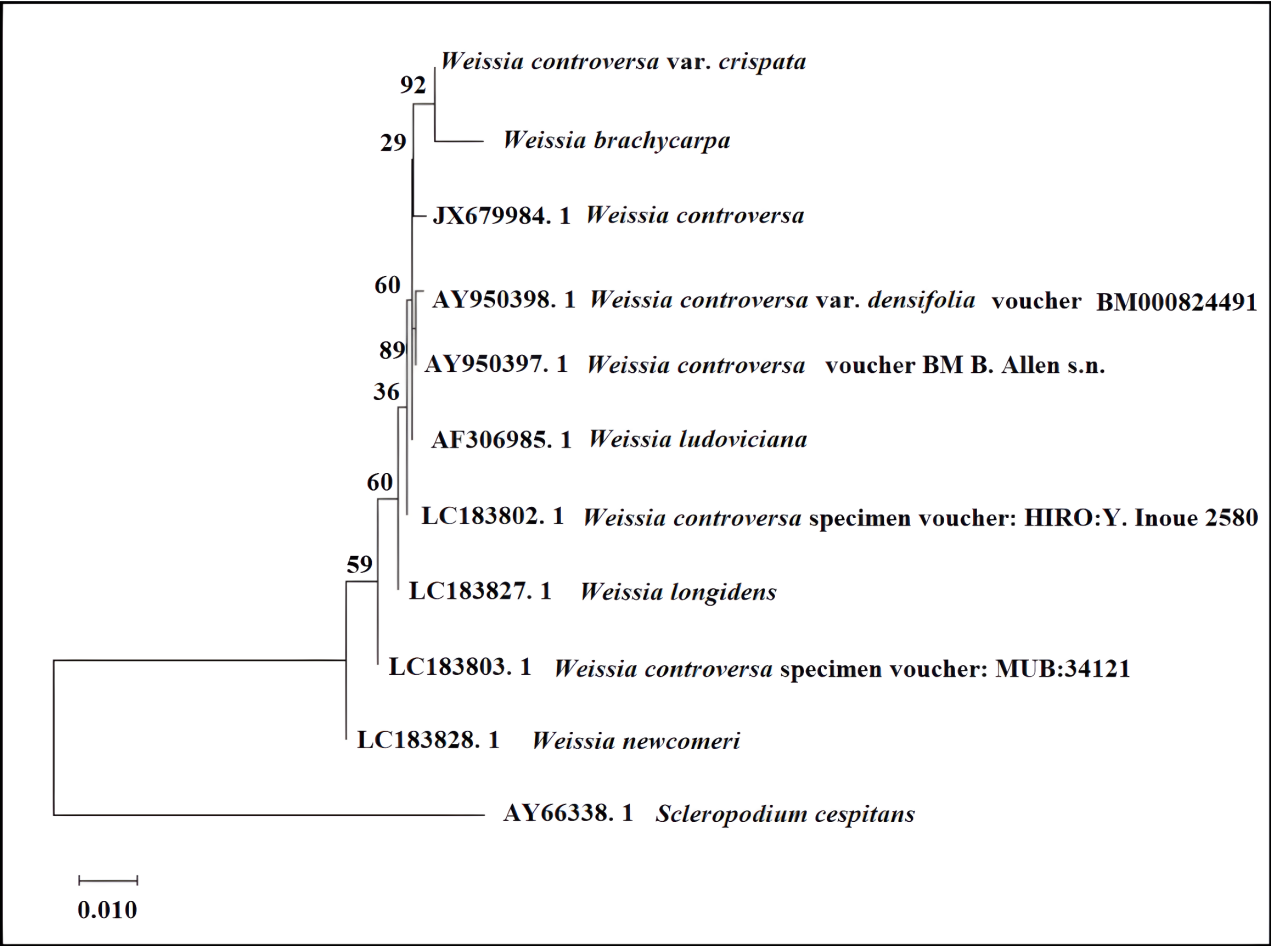
Phylogenetic tree of *Weissia* based on *psbA* gene. Phylogenetic tree of *Weissia* based on the *rps4* gene, inferred using the Neighbor-Joining method (MEGA X). Numbers above branches indicate bootstrap support values (%) from 1,000 replicates. The tree was rooted with *Scleropodium cespitans* as the outgroup.

In the rps4-based phylogenetic tree (Fig. 3), *W. brachycarpa* and *W. controversa* var. *crispata* formed a well-supported clade (bootstrap 92%). Other relationships within the *W. controversa* complex had relatively low bootstrap support (55–65%), indicating unresolved boundaries that require further study. Additionally, *W. newcomeri* appeared as a distinct lineage sister to the rest of the *Weissia* species, while *W. ludoviciana* was separated from *W. controversa* complex, supporting its taxonomic distinctness.

**Figure 4:**
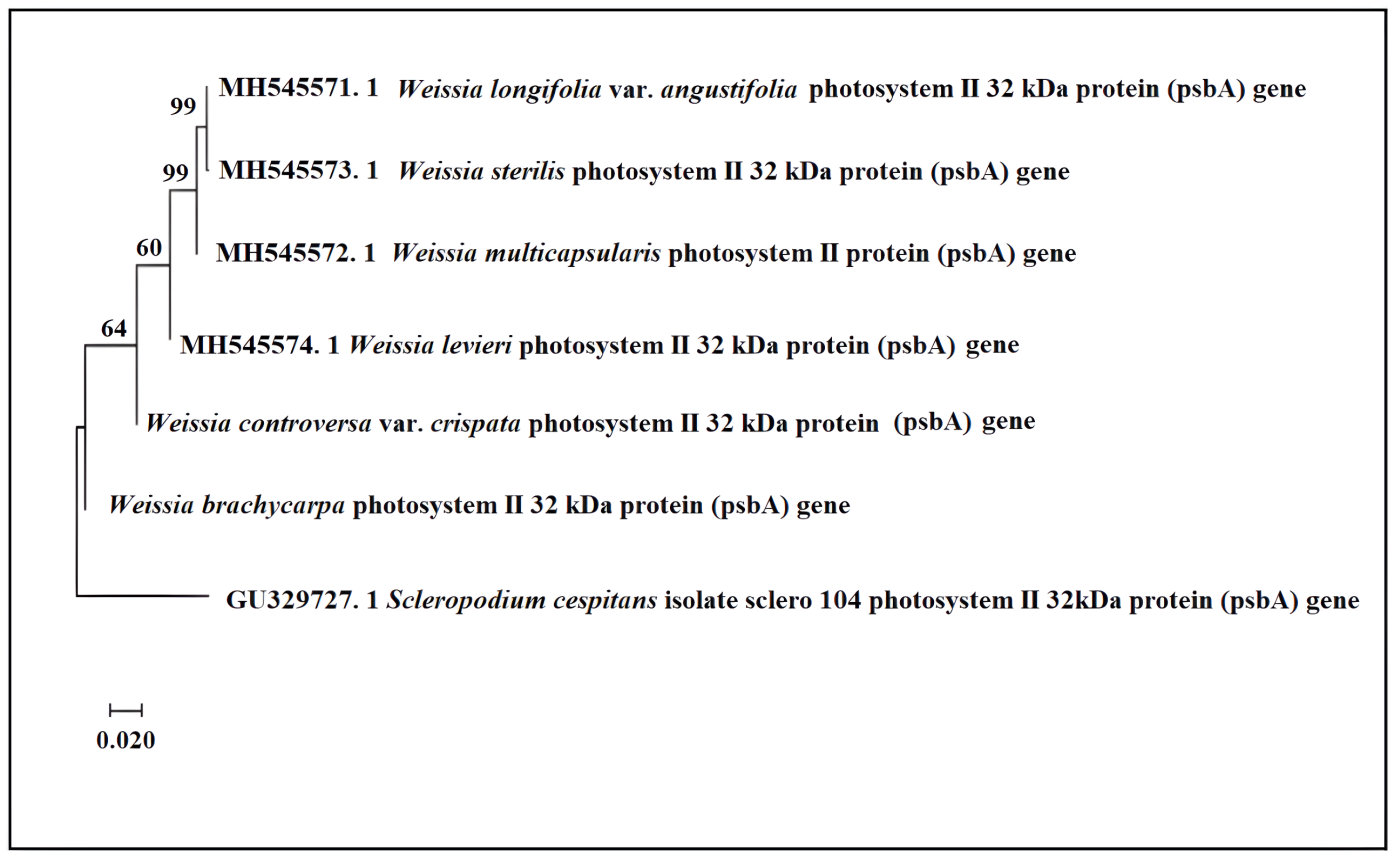
Phylogenetic relationships of *Weissia* based on *psbA* gene. Phylogenetic relationships of *Weissia* based on the *psbA* gene using the Neighbor-Joining method (MEGA X). Bootstrap support values (%) are shown at nodes. The analysis was rooted with *Scleropodium cespitans* as the outgroup.

In the *psbA*-based tree (Fig. 4), the relationship between *W. brachycarpa* and *W. controversa* var. *crispata* was not recovered. A well-supported clade (bootstrap 99%) united *W. longifolia* var. *angustifolia* with *W. sterilis*. In contrast, *W. multicapsularis* was sister to W. longifolia nad *W. sterelis*; while *W. levieri* was sister to all of them (∼60%). *W. condensa* and *W. rutilans* were not included in the psbA dataset.

**Figure 5:**
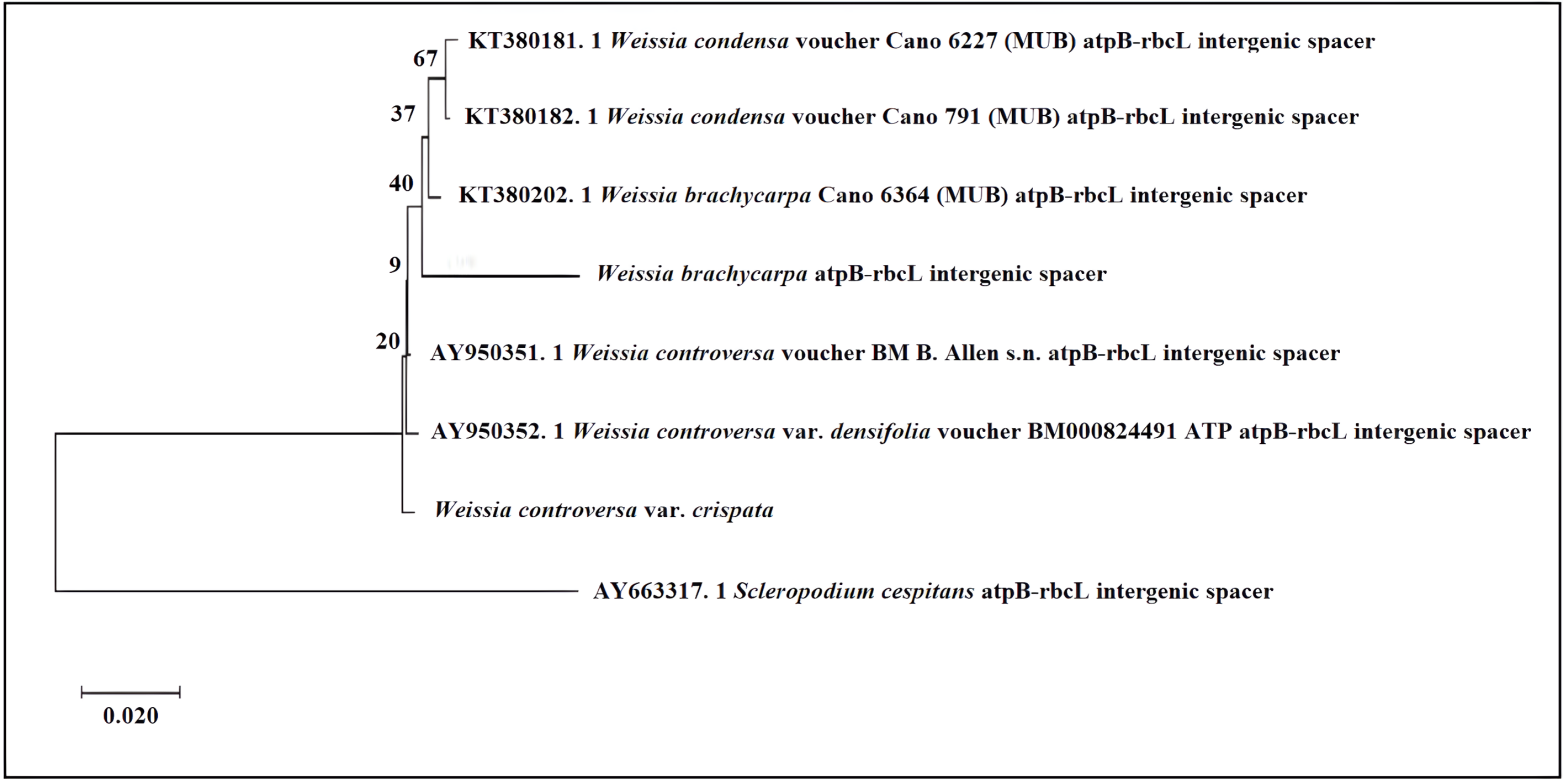
Phylogenetic relationships of *Weissia* based on *atpB-rbcL* intergenic region. Phylogenetic relationships of *Weissia* based on the *atpB-rbcL* intergenic spacer using the Neighbor-Joining method (MEGA X). Numbers above branches represent bootstrap support values (%) from 1,000 replicates. The tree was rooted with *Scleropodium cespitans* as the outgroup.

In the *atpB-rbcL* tree (Fig. 5), two accessions of *W. condensa* formed a moderately supported clade (bootstrap value 67%). The two accessions of W. *brachycarpa* were sister to *W. condensa*, but with low support (bootstrap value 40%). Members of the *W. controversa* complex (*W. controversa, W. controversa* var. *densifolia*, and *W. controversa* var. *crispata*) were sister to the previous taxa, but their relationship and position in the tree was unclear because of the very low support of the diverging nodes containing *W. controversa* and the remaining taxa (≤20), indicating limited resolution of the atpB-rbcL spacer and the need for additional loci.

## Discussion

### Morphological insights

The morphological variability observed among the *Weissia* taxa examined in this study suggests ecological adaptation to distinct microhabitats. For instance, *W. rutilans* exhibited the greatest plant height (up to 15 mm) and comparatively long perichaetia leaves (2–2.5 mm), likely supporting efficient spore dispersal in exposed environments. In contrast, *W. wilsonii* displayed the shortest vegetative leaves (0.8–2 mm) and the longest perichaetial leaves (up to 3.5 mm), reflecting potential adaptation to shaded, mesic habitats. Similar patterns of habitat-driven morphological adaptation are also reported in other mosses. For instance, cushion-forming species such as *Grimmia* develop dense tufts that reduce boundary-layer conductance and minimize water loss in xeric rocky environments (Bates, 1998; Smith, 2004), while pleurocarpous mosses like *Hypnum cupressiforme* produce elongated shoots and creeping stems that enhance light interception and moisture absorption in shaded forest floors (Glime, 2017). These examples illustrate how bryophyte morphology consistently reflects microhabitat demands. In *Weissia*, the relative size of perichaetial leaves appears to function similarly. Taxa from shaded or humid habitats (*e.g.*, *W. wilsonii*) develop disproportionately longer perichaetial leaves that elevate the capsule for effective dispersal, whereas species in open or drier sites (*e.g.*, *W. rutilans*) exhibit shorter but more rigid leaves that protect the sporophyte under fluctuating moisture conditions. Such examples reinforce how morphological traits in bryophytes are strongly shaped by microhabitat conditions (Rose, Kriebel & Sytsma, 2016).

Table 3 outlines key morphological comparisons among *Weissia* taxa. Critical traits including seta length, capsule size, peristome presence, and spore ornamentation proved essential for distinguishing morphologically similar species, such as *W. controversa* and *W. controversa* var. *crispata*. For example, although *W. brachycarpa* and *W. controversa* var. *crispata* share similar gametophytic features, our data indicate that *W. brachycarpa* typically exhibits shorter plant height (3–8 mm), shorter perichaetial leaves (2–3 mm), and lower ratios of perichaetial to vegetative leaf length (0.5–0.7), while *W. controversa* var. *crispata* possesses more robust sporophytes with perichaetial leaf lengths up to three mm and higher ratios of vegetative to perichaetial leaf length (2–3). These features, also reflected in the species key, enhance our ability to distinguish closely related taxa and reduce misidentification.

Additionally, the findings confirm that perichaetial leaf length and the ratio of perichaetial to vegetative leaf length are informative for distinguishing several *Weissia* species. For example, *W. longifolia* shows moderate leaf lengths (1–1.5 mm) with disproportionately large perichaetial leaves (2–5 mm), and *W. wilsonii* displays the shortest vegetative leaves but the longest perichaetial ones (up to 3.5 mm). In contrast, *W. condensa* and the *W. controversa* complex are more morphologically conservative, and this trait alone provides limited resolution for delimitating *W. condensa* nd *W. controversa*.

The ecological functions of *Weissia* species, particularly their ability to colonize disturbed or open soils, highlight their importance in early successional stages of bryophyte communities. For example, *W. controversa* often grows on compacted agricultural soils and roadside habitats, where it contributes to soil stabilization and erosion control (Gradstein, 1992; Slack, 1990). Similarly, *W. brachycarpa* has been reported on anthropogenic substrates such as paths and field margins, where its dense mats promote moisture retention and provide microhabitats for soil invertebrates (Zander, 1993). These examples demonstrate that *Weissia* species perform ecologically important functions yet several of these taxa are difficult to distinguish due to overlapping morphological traits. Because these ecosystem roles may differ among closely related species, resolving the taxonomic ambiguities within *Weissia* is essential for accurately assessing their functional significance and understanding how each taxon contributes to ecosystem dynamics.

Finally, the discovery of *W. wilsonii* in northwestern Türkiye extends the known distribution of this species, previously thought to be endemic to England. Its unique morphological profile, including perichaetial leaves up to 3.5 mm and very short setae (not shown in Table 3), along with its occurrence on moss-rich shaded rocks, suggests a broader ecological range. This record underscores the need for further biogeographic surveys and re-evaluation of conservation statuses for underreported bryophyte taxa in the region.

### Molecular phylogenetics

Our molecular analyses revealed marker-dependent genetic resolution among *Weissia* taxa. Some clades had weak to moderate support in the *psbA* and *atpB-rbcL* trees, showing that it’s difficult to confidently infer relationships for some species. The rps4 based tree showed the strongest support (bootstrap 92%) for the group containing *W. brachycarpa* and *W. controversa* var. *crispata*, indicating a close evolutionary relationship. In contrast, the *psbA* and *atpB-rbcL* trees provided weaker or mixed support; for instance, in the atpB-rbcL tree (bootstrap < 70%), bootstrap in *atpB*-*rbcL*), *W. brachycarpa* did not consistently cluster with *W. controversa* or its varieties, and members of the *W. controversa* complex appeared in poorly supported, scattered positions rather than forming a coherent clade. This inconsistency, also visible in the psbA tree where *W. brachycarpa* and *W. controversa* var. *crispata* failed to form a supported clade, highlights the marker-dependent instability of their inferred relationships. Such variation in phylogenetic placement suggests underlying taxonomic complexity within the *W. controversa* complex and may reflect hidden diversity, recent diversification events, or more complex evolutionary processes such as incomplete lineage sorting (Maddison, 1997; Shaw, Szövényi & Shaw, 2011). These patterns emphasize the need for more detailed studies using additional genomic markers and population-level sampling.

These findings partially align with Callaghan, Bell & Forrest (2019), who used ITS and chloroplast markers to show close relationship between *W. controversa* and *W. brachycarpa* populations in western Europe. However, their study did not include Turkish samples and reported stronger support in ITS-based trees compared to plastid data. Our results also show that plastid markers can uncover important relationships, but they can also lead to different conclusions depending on the marker used, especially in populations that are geographically isolated and present overlapping morphological attributes. In addition, Because *W. condensa* was included only in the *atpB-rbcL* tree and *W. longifolia* only in the psbA tree, their phylogenetic positions cannot be directly compared across markers. This lack of overlap illustrates a limitation of our dataset, highlighting the need to include more species and markers in future analyses to obtain a more comprehensive understanding of relationships within *Weissia*.

The phylogenetic tree created using the *rps4* gene in this study matches previous research and highlights how useful plastid genes are for understanding the evolutionary connections of *Weissia*, and potentially of higher-level moss classification. For instance, Werner, Ros & Grundmann (2005) demonstrated that the *rps4* marker offers strong resolution at the species level and is effective in delineating evolutionary relationships among mosses. In particular, Inoue & Tsubota (2017) showed that new molecular data, specifically a combined plastid dataset consisting of the rbcL and rps4 gene regions, led to taxonomic revisions within Japanese *Weissia*, resulting in several species being reclassified or synonymized based on phylogenetic evidence. These findings illustrate that plastid markers such as *rps4* not only clarify relationships within *Weissia* but also contribute to ongoing efforts to stabilize bryophyte taxonomy at a broader scale. Our results support these findings by showing that *W. brachycarpa* and *W. controversa* var. *crispata* are closely related in the *rps4* tree and confirming that this marker is reliable for distinguishing between these two species. This relationship is also supported by morphological similarities, as both taxa share overlapping leaf dimensions and capsule characters; however, subtle differences such as the crispate leaf margins in *W. controversa* var. *crispata* remain consistent diagnostic features.

The phylogenetic tree created using the *psbA* gene strongly supports several clades, like the connection between *W. longifolia* var. *angustifolia* and *W. sterilis*, which has a bootstrap value of 99%. These species also exhibit similar morphological traits, such as leaf shape and spore size, suggesting that the genetic relationships observed in the *psbA* tree partially correlate with morphological data. As Callaghan, Bell & Forrest (2019) noted, plastid markers like *psbA* are invaluable for detecting cryptic species and understanding patterns of evolutionary divergence. This is partly because plastid genes evolve more slowly than nuclear ones, due to their uniparental inheritance, lack of recombination, and stronger functional constraints on photosynthetic proteins (Shaw, Szövényi & Shaw, 2011; Stech & Frey, 2008). These features make plastid markers particularly reliable for resolving higher rank relationships, although they may be less informative for recent or rapidly evolving lineages. For instance, in the *atpB-rbcL* tree, *W. condensa* and *W. brachycarpa* form a moderately supported clade, rather than occupying distinct positions. This pattern may reflect partial plastid-based affinity despite some morphological differences and should be tested with additional loci and broader sampling. These findings emphasize the role of both ecological context and evolutionary history in shaping phylogenetic outcomes.

Research on (*e.g.*, *Weissia* and *Scleropodium*) has shown that the *atpB–rbcL* intergenic spacer is informative for resolving phylogenetic relationships, primarily because its non-coding regions exhibit substantial nucleotide variation (*e.g.*, Werner, Ros & Grundmann, 2005; Inoue & Tsubota, 2017). The evolutionary signal of this marker is generally informative at the species level or higher rank; however, its slower substitution rate can limit resolution of very recent divergences, so plastid data should be interpreted alongside nuclear loci (Inoue & Tsubota, 2017; Callaghan, Bell & Forrest, 2019). In our study, however, the *atpB-rbcL* tree exhibited low to moderate bootstrap values for several clades, suggesting that while this marker can help identify relationships, its resolution may be limited in some *Weissia* lineages without additional loci or broader taxon sampling.

Nevertheless, the low bootstrap support we observed in our analysis is consistent with the findings of Werner, Ros & Grundmann (2005), who reported weak resolution in some deep-level relationships within Pottiaceae when using the *atpB–rbcL* spacer. Although this marker has proven useful for resolving deeper phylogenetic patterns in several studies, including within Pottiaceae, its performance can vary among lineages and may be insufficient on its own for reconstructing complex or recently diverged clades. This highlights the importance of integrating multiple genetic markers and broader taxon sampling for robust phylogenetic inference (Inoue & Tsubota, 2017).

### Limitations and future directions

Although this study offers valuable insights, it is important to acknowledge certain limitations. First, only three plastid markers (*rps4, psbA*, and *atpB–rbcL*) may not adequately resolve certain evolutionary relationships, particularly among clades with weak bootstrap support. Incorporating nuclear markers in future studies could offer better resolution and uncover potential hybridization events, as nuclear genes evolve independently from plastid DNA and exhibit higher recombination rates and biparental inheritance. This provides complementary information that can help detect reticulate evolution and clarify relationships obscured by plastid data alone. Second, the number of available specimens per species was limited, and some taxa were represented by single collections, which may not fully capture intraspecific variation. Third, relying on herbarium material instead of fresh collections could potentially introduce biases related to DNA degradation. Expanding the sampling both geographically and taxonomically, along with applying genomic approaches, will be essential to resolve the remaining taxonomic uncertainties within the genus *Weissia*.

## Conclusion

This study provides a combined morphological and molecular analysis of *Weissia* taxa in Türkiye, offering insights into their phylogenetic relationships, species diversity, and morphological variability. The results highlight the genetic distinctiveness of *W. brachycarpa*, *W. controversa* var*. crispata*, and *W. condensa* while suggesting potential taxonomic complexities within the *W. controversa* group. The congruence observed between morphological traits and molecular data underscores the importance of combining these approaches to achieve a more comprehensive understanding of *Weissia* taxonomy.

The plastid markers *rps4, psbA*, and the *atpB-rbcL* intergenic spacer were used to understand the evolutionary relationships and genetic differences within the genus *Weissia*. The *rps4*-based phylogeny, for instance, strongly supported a close evolutionary relationship between *W. brachycarpa* and *W. controversa* var. *crispata* (bootstrap 92%), while other markers showed weaker or inconsistent resolution among the same taxa. These patterns suggest possible lineage divergence events, potentially shaped by environmental heterogeneity or geographic isolation. Nevertheless, the presence of low bootstrap values in several clades, particularly in the *psbA* and *atpB-rbcL* trees, indicates that the current plastid data alone are insufficient to fully resolve species boundaries and their evolutionary relationships. Additional molecular markers, especially nuclear loci, along with broader taxon sampling, are needed to clarify the evolutionary trajectories and potential speciation processes within *Weissia*.

This research contributes to the regional understanding of *Weissia* diversity and evolution while providing a foundation for future conservation strategies, especially in taxa that was not recorded before, such as *W. wilsonii*. The ecological significance of *Weissia* taxa, particularly in soil stabilization and microhabitat formation, emphasizes the need to preserve their habitats.

Despite clarifying relationships among several *Weissia* taxa, questions remain regarding the placement of *W. rutilans* and the broader intraspecific variation in *W. controversa*. Future studies should include nuclear loci (*e.g.*, ITS) and employ genome-scale techniques such as target enrichment or genome skimming. Expanded sampling from other regions of Türkiye and neighboring countries may also improve our understanding of biogeographic patterns and unresolved lineages.

### Key to *Weissia* species in Türkiye

**Table:** 

1 Capsule immersed ……………………………………………………...........................2
1 Capsule exposed ………………………………………………………………..……3
2 Seta length >1 mm and leaf basal cells length <40 µm ..……………...*W. longifolia*
2 Seta length >1 mm and leaf basal cells length >40 µm ………………*W. wilsonii*
3 Plant length >8 mm long………………………..…….................…*W. rutilans*
3 Plant length <8 mm long …………………………………..............................4
4 Peristome teeth present ………………………...................................... ………….5
4 Peristome teeth apsent ……………………………………………………...........6
5 Seta >2 mm long and spore papillose …………………….......... *W. controversa*
5 Seta <2 mm long and spore smooth to slightly papillose ................………………................ ……….................... *W. controversa* var. *crispata*
6 Leaf base costa thickness >55 µm and leaf apical cell <16 µm …………. *W. condensa*
6 Leaf base costa thickness <55 µm and leaf apical cell >16 µm....…. *W. brachycarpa*

##  Supplemental Information

10.7717/peerj.20967/supp-1 Supplemental Information 1 Alignment of sequences for *atpB*-*rbcL* intergenic spacer

## Additional Information and Declarations

### DNA Deposition

The following information was supplied regarding the deposition of DNA sequences: Nucleotide sequences are available at GenBank: PQ821690 and PQ821691 for rps4, PQ821692 and PQ821693 for psbA, and PQ821694 and PQ821695 for atpB-rbcL.

### Data Availability

The following information was supplied regarding data availability: The data is available in the Supplemental Files.
